# Formalin-casein enhances water absorbency of calcium alginate beads and activity of encapsulated *Metarhizium brunneum* and *Saccharomyces cerevisiae*

**DOI:** 10.1007/s11274-021-03121-3

**Published:** 2021-08-18

**Authors:** Katharina M. Hermann, Alexander Grünberger, Anant V. Patel

**Affiliations:** 1 Faculty of Engineering and Mathematics, Fermentation and Formulation of Biologicals and Chemicals, Bielefeld University of Applied Sciences, Bielefeld, Germany; 2 Faculty of Technology, Multiscale Bioengineering, Bielefeld University, Bielefeld, Germany

**Keywords:** Alginate beads, Attract-and-kill, Formalin-casein, *Metarhizium brunneum*, Porosity, Water absorption, *Saccharomyces cerevisiae*

## Abstract

The control of root-feeding wireworms has become more challenging as synthetic soil insecticides have been progressively phased out due to environmental risk concerns. Innovative microbial control alternatives such as the so-called attract-and-kill strategy depend on the rapid and successful development of dried encapsulated microorganisms, which is initiated by rehydration. Casein is a functional additive that is already used in food or pharmaceutical industry due to its water binding capacity. Cross-linked forms such as formalin-casein (FC), exhibit altered network structures. To determine whether FC influences the rehydration of alginate beads in order to increase the efficacy of an attract-and-kill formulation for wireworm pest control, we incorporated either casein or FC in different alginate/starch formulations. We investigated the porous properties of alginate/starch beads and subsequently evaluated the activities of the encapsulated entomopathogenic fungus *Metarhizium brunneum* and the CO_2_ producing yeast *Saccharomyces cerevisiae*. Adding caseins altered the porous structure of beads. FC decreased the bead density from (1.0197 ± 0.0008) g/mL to (1.0144 ± 0.0008) g/mL and the pore diameter by 31%. In contrast to casein, FC enhanced the water absorbency of alginate/starch beads by 40%. Furthermore, incorporating FC quadrupled the spore density on beads containing *M. brunneum* and *S. cerevisiae*, and simultaneous venting increased the spore density even by a factor of 18. Moreover, FC increased the total CO_2_ produced by *M. brunneum* and *S. cerevisiae* by 29%. Thus, our findings suggest that rehydration is enhanced by larger capillaries, resulting in an increased water absorption capacity. Our data further suggest that gas exchange is improved by FC. Therefore, our results indicate that FC enhances the fungal activity of both fungi *M. brunneum* and *S. cerevisiae*, presumably leading to an enhanced attract-and-kill efficacy for pest control.

**Graphic abstract:**

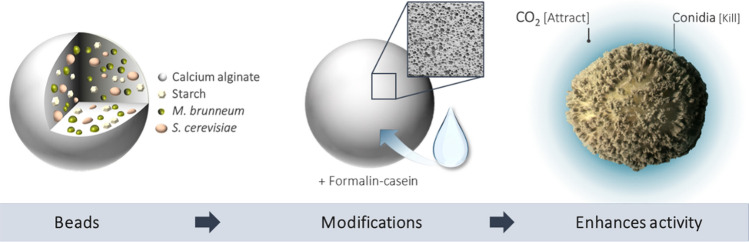

**Supplementary Information:**

The online version contains supplementary material available at 10.1007/s11274-021-03121-3.

## Introduction

Wireworms (*Coleoptera*: *Elateridae*) are widespread pests that damage a variety of crops, including grain i.e. wheat, barley, maize and most vegetables such as carrot and potato. Since the mid-twentieth century wireworm damage is becoming an increasing concern in both conventional and organic potato cultivation (Poggi et al. 2018; Vernon and van Herk 2013). Wireworms can be very destructive and difficult to control. Even low populations lead to reduced quality of the harvested tubers and thus to severe economic losses (Parker and Howard 2001). Effective plant protection products are currently not available, since chemical insecticides have been restricted or abandoned. Neonicotinoids and fipronil were the main classes of insecticides routinely used and both have been restricted by the European Union (European Food Safety Authority 2013). Consequently, there is a tremendous need for control options, either conventional, or better yet, biological such as microbial pest control.

There are several approaches to microbial pest control, one of which is the attract-and-kill strategy, where pests are attracted toward an entomopathogenic pathogen (Brandl et al. 2017). Humbert et al. (2017a, b) and Pryzyklenk et al. (2017) developed an alginate bead formulation containing the CO_2_ producing yeast *Saccharomyces cerevisiae* and the entomopathogenic fungus *Metarhizium brunneum* (Humbert et al. 2017a, b; Przyklenk et al. 2017). Native corn starch is additionally incorporated into these beads as an indispensable filler material and nutrient supply. *M. brunneum* produces amylolytic enzymes and is therefore able to degrade starch, providing glucose for both fungi (Przyklenk et al. 2017). The attract-and-kill approach takes advantage of the behavior of wireworms and other soil dwelling larvae which use CO_2_ for locating growing roots of potential host plants (Arce et al. 2021). Dried beads are put below ground whereupon they absorb soil moisture, reactivating the encapsulated microorganisms. As a result, wireworms are attracted by the CO_2_ and are subsequently infected by the virulent aerial conidia of *M.* *brunneum,* formed on the bead surface (Brandl et al. 2017; Schumann et al. 2014). With this approach, the onset of action and the effect itself depend on the development and activity of both fungi. The development of the fungi, in turn, depends on sufficient rehydration of the dried formulation. Therefore, adequate water absorption of alginate beads and eventual water retention is crucial for a certain and reliable efficacy.

Three major approaches to modify the water absorbency of alginate gels have already been studied: I. The choice of crosslinking agents and the crosslinking time (Kim et al. 2000; Kulkarni et al. 2000), II. the enzymatic *in-vitro* modification of alginate (Mørch et al. 2007), and III. blending other materials such as biopolymers (Chan et al. 2008).

Biopolymers have been investigated for their capability of enhancing the water absorbency specifically of calcium alginate beads. In studies by Vreeker et al. (2008) and Fang et al. (2011) beads with gelatin, gum arabic or carboxymethylcellulose rehydrated faster. The polymers probably altered the supramolecular structure of the alginate gel during drying. More specifically, they might reduce the side-by-side aggregation of the alginate egg box junctions, resulting in less dense beads. However, as major drawback, gum arabic only reached the equilibrium swelling ratio of control alginate beads, and gelatin even decreased it. For carboxymethylcellulose, equilibrium was not even determined (Fang et al. 2011; Vreeker et al. 2008).

However, proteins generally inhere excellent functional properties and are commonly used in food and pharmaceutical industry because of their high nutritional value and emulsification, gelation, foaming and water binding capacity (Elzoghby et al. 2011; Zayas 1997). Casein, the major milk protein, is generally regarded as safe, readily available, comparatively inexpensive, non-toxic and biodegradable. Casein can be crosslinked through chemical agents such as formaldehyde, resulting in a more complex network structure and altering its mechanical properties (Elzoghby et al. 2011). The resultant formalin-casein (FC) has proven to be suitable as non-swelling tablet-disintegrant (Fekete et al. 1990; Ponchel and Duchene 1990). Nevertheless, there have been only few reports on alginate/casein hydrogel systems, which were investigated for their mechanical, release, or physico-chemical properties (Bajpai et al. 2017; He et al. 2015; Patwa et al. 2020). Patwa et al. (2020) demonstrated that amide cross-linked alginate–casein hydrogels for wound healing applications, exhibited fast swelling with high water uptake.

To the best of our knowledge no studies have been reported yet on incorporating casein or FC into calcium alginate which already contained starch as filler. Hence, the main objective of this study was to investigate how FC impacts the water absorbency of calcium alginate/starch formulations, used for attract-and-kill pest control, and whether this, in turn, enhances its effectiveness. Although starch is already included in the formulation, acting as spacer between alginate junctions, we aimed to use FC as rehydration additive to further enhance water absorbency.

The first part of this work elucidates the influence of casein and FC on the porous properties and the rehydration of alginate/starch beads. The second part clarifies how casein and FC, and consequently the bead properties, influence the fungal activity of *M. brunneum* in Kill beads or of *S. cerevisiae* and *M. brunneum* in attract-and-kill beads (A&K beads), aiming to quickly provide a sufficient amount of attractive CO_2_ and entomopathogenic conidia.

## Methods

### Materials

All chemical compounds used in this study were acquired from Carl Roth GmbH (Karlsruhe, Germany) and concentrations are given as (w/w), unless stated otherwise.

### Preparation of Caseins

Casein from bovine milk (Sigma Aldrich, USA) was grinded in a ball mill and sieved subsequently to reach a particle size ≤ 150 µm. Formalin-casein (FC, commercialized as EsmaSpreng, Gaerungschemie Dessau GmbH, Dessau, Germany) was supplied in the appropriate particle size and was therefore only sieved to select particles ≤ 150 µm.

### Microorganisms and culture conditions

*S. cerevisiae* H205 was provided ready for use by Deutsche Hefewerke GmbH (Nürnberg, Germany).

*M. brunneum* strain CB15 was obtained from Prof. Dr. Stefan Vidal (Agricultural Entomology, Department for Crop Science, Georg-August-University Goettingen, Germany). To produce aerial conidia (AC) used for inoculation of submerged cultures, freeze-dried biomass was thawed in Potato Dextrose Broth (PDB) and cultivated on Potato Dextrose Agar (PDA) in the dark at 25 °C for 7 days. Plates were sealed with Parafilm M (Pechiney Plastic Packaging Inc., IL, USA) to avoid moisture loss. Then, the pre-culture was inoculated with AC resulting in a final concentration of 1 × 10^6^ AC/mL and cultivated in liquid medium modified according to Krell et al. (2018) (7.5% glucose*,* 4.0% ANiPept (ANiMOX GmbH, Berlin, Germany, batch No. 1176), 7.0% polyethylene glycol 200) at 25 °C and 150 rpm in 250 mL baffled shake flasks for 48 h. Cultures were inoculated with 5.0% (v/v) vegetative pre-culture and cultivated for 72 h. The mycelial biomass was harvested by vacuum filtration through filter paper (Whatman No. 4, qualitative, 20–25 μm pore size) and rinsed with 0.9% NaCl solution to remove blastospores and residual cultivation medium.

### Bead production

The preparation of calcium alginate beads was carried out with sterilized materials under aseptic conditions. Prior to encapsulation, sodium alginate (CEROGA Sodium Alginate Type NA5030, C.E. Roeper GmbH, Hamburg, Germany) was dissolved in ultrapure water to a final concentration of 4.0% and autoclaved for 6 min at 121 °C. The encapsulation suspensions were prepared as follows.

#### Calcium alginate/starch beads

The encapsulation suspension for the control beads was produced by thoroughly mixing 2.0% calcium alginate with 20.0% native corn starch (CIF GmbH, Siegburg, Germany) for 10 min until suspended. Finally, water was added to reach final concentrations.

Encapsulation suspension with casein additives was produced by mixing 2.0% sodium alginate with 13.3% native corn starch for 5 min and then with 6.7% either casein or formalin-casein for 5 min until suspended. Because the suspension became viscous, the water required to reach final concentrations was added shortly before dripping.

#### Kill beads

The encapsulation suspension for the control Kill beads was produced by mixing 2.0% sodium alginate with 20.0% native corn starch for 10 min until suspended. Then, 1.0% *M. brunneum* mycelial biomass was added and gently stirred for 2 min. Water was added to reach the final concentrations.

The encapsulation suspension with either casein or FC was produced by mixing 2% sodium alginate with 13.3% native corn starch for 5 min, and subsequently with 6.7% either casein or formalin-casein for 5 min until suspended. Then, 1.0% *M. brunneum* mycelial biomass was added and gently stirred for 2 min. The water required to reach final concentrations was added shortly before dripping.

#### Attract-and-kill beads

The encapsulation suspension for the control A&K beads was produced by mixing 2.0% sodium alginate with 16.7% *S. cerevisiae* for 5 min until blended and then with 20.0% native corn starch for 10 min. Then, 1.0% *M. brunneum* mycelial biomass was added and gently stirred for 2 min. Finally, water was added to reach final concentrations.

The encapsulation suspensions with casein or FC were prepared the same way, but only 13.3% native corn starch was mixed for 5 min before 6.7% either casein or FC was added and mixed for another 5 min.

#### Bead formation

Beads were prepared by dripping the suspension into a sterile 180 mM CaCl_2_ solution by means of a single syringe pump (Cole-Parmer, Vernon Hills, USA), with a 0.9 mm cannula (Sterican, B. Braun Melsungen AG, Melsungen, Germany). The pumping speed was set to 4 mL/min. After 20 min, beads were separated and washed with ultrapure water. Moist beads were dried under a laminar flow at room temperature (23 °C, 30% relative humidity) for 24 h. Alginate/starch beads were subsequently dried over silica to reach a final water activity of 0.2, unless specified otherwise. Water activities were controlled with a water activity meter (LabMASTER-aw, Novasina AG, Lachen, Switzerland) at 25 °C.

### Analysis of calcium alginate/starch beads

#### Determination of C/N ratio

The C/N ratio of alginate beads was analyzed with a C/N elemental analyzer (Unicube®, temperature programmed desorption column, Elementar, Langenselbold, Germany) to assess the nutritive influence of casein and FC. Dried calcium alginate beads with starch or starch and either casein or FC were grinded with a ball mill to obtain a homogenous composition of the sample matrix. The amount of 5 g of each matrix was weighed in tin containers and loaded into an automatic sampler. Each sample matrix was measured twice.

#### Determination of bead size, bead count and bead shape

The particle projected area diameters derived from digital images of the beads were determined using the open source image processing program Fiji (ImageJ, U. S. National Institutes of Health, Bethesda, USA) (Schindelin et al. 2012). For this, beads were placed in the center of a Petri dish on a black background and images including a reference scale were taken using a tripod. For following batch analysis by help of a macro (Online Resource 1, Fig. A1), images were taken at the same magnification. The parameters of the macro were adapted for the respective batch. The determined diameters were automatically exported in an excel file and used to calculate the bead surface area. Beads were counted simultaneously to bead size measurements. Additionally, the bead shape of dried and rehydrated beads (after 48 h) was determined, which is represented by the circularity and the sphericity factor of the particle projected area. The latter was determined according to Chan et al. (2011).

$$Sphericity \, factor = \frac{{d_{max} - d_{min} }}{{d_{max} + d_{min} }}$$

where $d_{max}$ is the largest diameter and $d_{min}$ is the smallest diameter perpendicular to $d_{max}$.

#### Determination of water absorption and water activity

Rehydration was measured as a function of time according to Vreeker et al. (2008).

In preliminary investigations we found that the rehydration of beads reaches its maximum within 48 h. Based on these findings, we chose to examine the rehydration within this period more closely. Therefore, the water absorption of beads was determined gravimetrically in defined time intervals between 0 min and 48 h.

Approximately 100 dried beads were placed on 15 mL 1.5% water agar in closed Petri dishes. For each time point separate Petri dishes were prepared in three replicates. The exact number of beads of each sample was determined by digital image analysis using the open source image processing program Fiji (see ‘Determination of bead size, bead count and bead shape’) and was later set in relation to the measured sample weight.

Beads were withdrawn from water agar and excess water was carefully removed by blotting on lint-free tissue before weighing on a precision balance (VWR, Pennsylvania, USA) in small, closed Petri dishes. Rehydration $R\left( t \right)$ is expressed as

$$R\left( t \right) = \frac{{m\left( t \right) - m_{d} }}{{m_{0} - m_{d} }}$$

where $m\left( t \right)$ represents the mass of beads during rehydration; $m_{0}$ is the mass of fresh beads before drying and $m_{d}$ is the mass of dried beads.

Afterwards the samples were transferred into special airtight containers for water activity measurements and stored until measured. Water activities were determined with a water activity meter (LabMASTER-aw, Novasina AG, Lachen, Switzerland) at 25 °C. Water activity and temperature stable time were set to 2 min and 1 min, respectively.

#### Determination of bead density

The bead density $\rho_{p}$ of the different calcium alginate/starch formulations with either casein or FC was calculated using the settling velocity ($v$) in water according to Stokes equation.

$$v = \frac{{\left( {\rho_{p} - \rho_{f} } \right) \cdot \overline{d}^{2} \cdot g}}{18 \cdot \eta }$$

where $\rho_{f}$ is the density of water, $d$ is the mean bead projected area diameter, which was determined for 50 beads (see ‘Determination of bead size, bead count and bead shape’), $g$ is the gravity with 9.81 m/s, $\eta$ represents the dynamic viscosity of water and is 0.000931 kg/m s. Digital video recordings of the beads at 120 fps were taken and the settling velocity was determined with an open source video editing program (VideoPad Video Editor Free, NCH Software). For each kind of formulation 20 beads were analyzed.

#### Determination of pore volume distribution and pore size

We used differential scanning calorimetry (DSC) to assess the pore volume distributions and therefore how casein or FC influence the pore sizes in calcium alginate/starch beads. DSC measurements were modified according to the measurements of pore sizes in silica gels by Kazuhiko Ishikiriyama et al. (1995a, b) and Maloney (2000) using a Mettler Toledo DSC-3 device.

Dried beads were rehydrated on water agar in Petri dishes and then immersed in excessive water overnight to remove residual ions and to fill the pores with water. Accurately weighed samples (3–6 mg) were sealed in 40 µL aluminum pans with pins. Because of their weight and size, rehydrated beads needed to be cut to fit into the aluminum pans. The samples were measured against an empty reference pan. Nitrogen served as purge gas at 50 ml/min. The measurements were performed over a temperature range from 233.15 to 283.15 K and melting curves were measured to determine the pore volume distributions. Samples were rapidly cooled to 233.15 K, held isothermally for 30 min, and subsequently heated to 183.15 K at a low scanning rate of 0.5 K/min to avoid thermal and time delays. After DSC measurements, samples were dried at 60 °C for 3 days and the dry weight or rather the mass of the water was determined. Measurements were conducted three times with three different beads for each kind of formulation. Resulting peaks were analyzed according to Maloney (2000) with the STARe evaluation software (Version 15, Mettler Toledo, Columbus, USA) and transformed into pore volume distributions using Microsoft Excel (Microsoft Corporation, Washington, USA). The relationship between the pore diameter $D$ and the melting point depression Δ*T*_*m*_ is described by the Gibbs -Thomson equation:

$$D = \frac{{4 \cdot V_{m} \cdot T \cdot \sigma_{ls} }}{{\Delta H_{m} \cdot \Delta T_{m} }}$$

$V_{m}$ is the mol volume of the used absorbate, in this case, water with 19.6 10^–6^ m^3^/mol, $T$ is the melting point of water 273.14 K, $\sigma_{ls}$ represents the calcium alginate and water boundary tension, which was previously determined by contact angle measurement using the sessile drop technique (OCA 15EC model, SCA 20 control software module, DataPhysics Instruments GmbH, Filderstadt, Germany) and resulted in 78.44 mN/m. Δ*H*_*m*_ is the heat of fusion of water which is 6.02 kJ/mol.

The total volume $V$ of the pores with the relevant diameter can be determined from the peak area (heat fusion $H_{I}$) measured at the according temperature

$$V = \frac{{H_{I} }}{{\left( {\rho_{w} \cdot \Delta H_{s} } \right)}}$$

$\rho_{w}$ represents the density of water or ice at the corresponding temperature. Δ*H*_*s*_ is the temperature dependent specific heat of fusion of the ice-water transition concerning freezable pore water which was determined according to Ishikiriyama et al. (1995a, b) using the equation of Randall (National research council of the United States of America 1930) in J/g where $\Delta T_{m}$ is the depression of the melting temperature of water.

$$\Delta H_{s} \; = \;334.1\; + \;2.119 \cdot \Delta T - 0.00783 \cdot \Delta T$$

The layer thickness of the non-freezable water was neglected.

### Assessment of the fungal activities

#### Determination of the spore density on beads

The spore forming activity of *M. brunneum* on Kill and A&K beads was determined by counting the conidia formed on the bead surface using a cell counting chamber (Thoma new, Paul Marienfeld GmbH & Co. KG, Lauda-Königshofen, Germany).

Beads were produced as described previously (see ‘Bead production’) with biomass from the same culture. Dried beads were rehydrated and eventually incubated on 1.5% water agar in sealed Petri dishes at 25 °C in the dark. The spore density was determined in defined time intervals within 14 days. At each sampling point and for each kind of formulation, ten beads were analyzed. Each bead was transferred into 1 mL 0.1% Tween80 and thoroughly mixed (Vortex Genie, neoLab Migge GmbH, Heidelberg, Germany) for at least 1 min to detach the spores. To calculate the spore density, the bead surface area was determined from the projected area diameter (see ‘Determination of bead size, bead count and bead shape’) of 100 rehydrated beads per formulation.

The spore density was evaluated again after 21 days, in order to compare the sporulation with that in a vented system (see ‘CO_2_ release measurements from beads’). It should be noted that over time mycelial growth was favored and spore densities may be underestimated, since spores adhered in the dense mycelium.

#### CO_2_ release measurements from beads

To assess how casein and FC influenced the metabolic activity of *S. cerevisiae* and/or *M. brunneum*, the amount of CO_2_ released from dried and rehydrated beads was measured using a carbon dioxide meter with pump aspirated sampling (CARBOCAP® GM 70; Vaisala Oyj, Helsinki, Finland).

For this purpose, 1 g of dried alginate/starch beads, Kill beads or A&K beads with or without either casein or FC were rehydrated and eventually incubated at room temperature in the dark on 5 mL water agar in sterile glass bottles with a defined volume of 134.4 mL. The glass bottles were vented with sterile pressured air to avoid CO_2_ accumulation and inhibition during cultivation. Additionally, the pressured air was humified using a gas washing bottle to prevent the beads and the water agar from drying. The relative humidity reached approximately 80%. By means of a compressed air distributor, 18 vessels were vented and cultured at once (Fig. 6a). The air flow rate was set to 15 L/h (equals one volume exchange per 10 min) using a variable area flowmeter.

In order to determine the CO_2_ productivity, the initial CO_2_ concentration was measured before the glass bottles were closed for 60 min. The in- and outlets of the carbon dioxide meter pump were connected to the glass bottles via sterile filters, thus the air circulated during the measurement. Each sample was measured for 2 min. Subsequently, the device was conditioned with ambient air for at least 1 min. The amount of released CO_2_ was measured every day over a period of 20 days and was converted from ppm to mL CO_2_ produced per g dried beads in 1 h. All measurements were performed in three replicates (three glass bottles) simultaneously. The biomass from the same culture or batch was used for each kind of formulation. The spore density on beads was determined after the experiment was terminated, i.e. after 21 days.

The CO_2_ productivity of control alginate/starch beads without *M. brunneum* or *S. cerevisiae* biomass, was previously evaluated and was negligible low (Online Resource 1, Fig. A2) and therefore, in subsequent experiments neglected. Due to surprising results, the experiment was repeated for Kill beads and A&K beads with and without FC.

**Fig. 1:**
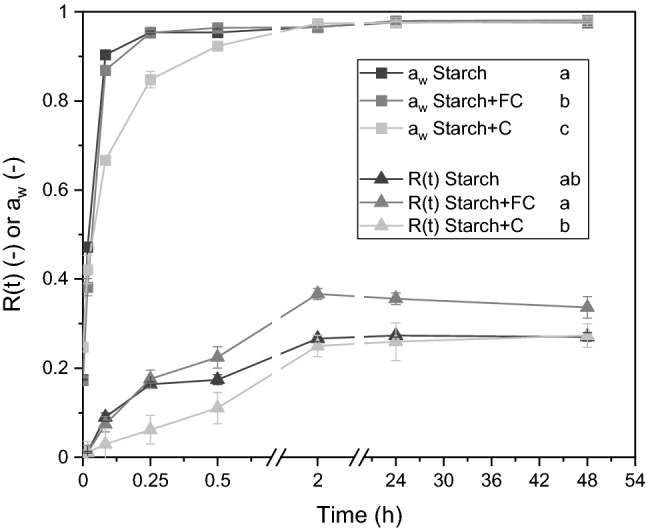
The maximum water absorption but not the water activity of alginate/starch beads was enhanced by formalin-casein (FC). The rehydration factor and the water activity of alginate/starch beads containing casein (C) or FC were rehydrated on 1.5% water agar at room temperature. Different letters next to the legend description indicate significant differences according to RM-ANOVA with Bonferroni post-hoc test at P < 0.05 (n = 3)

**Fig. 2:**
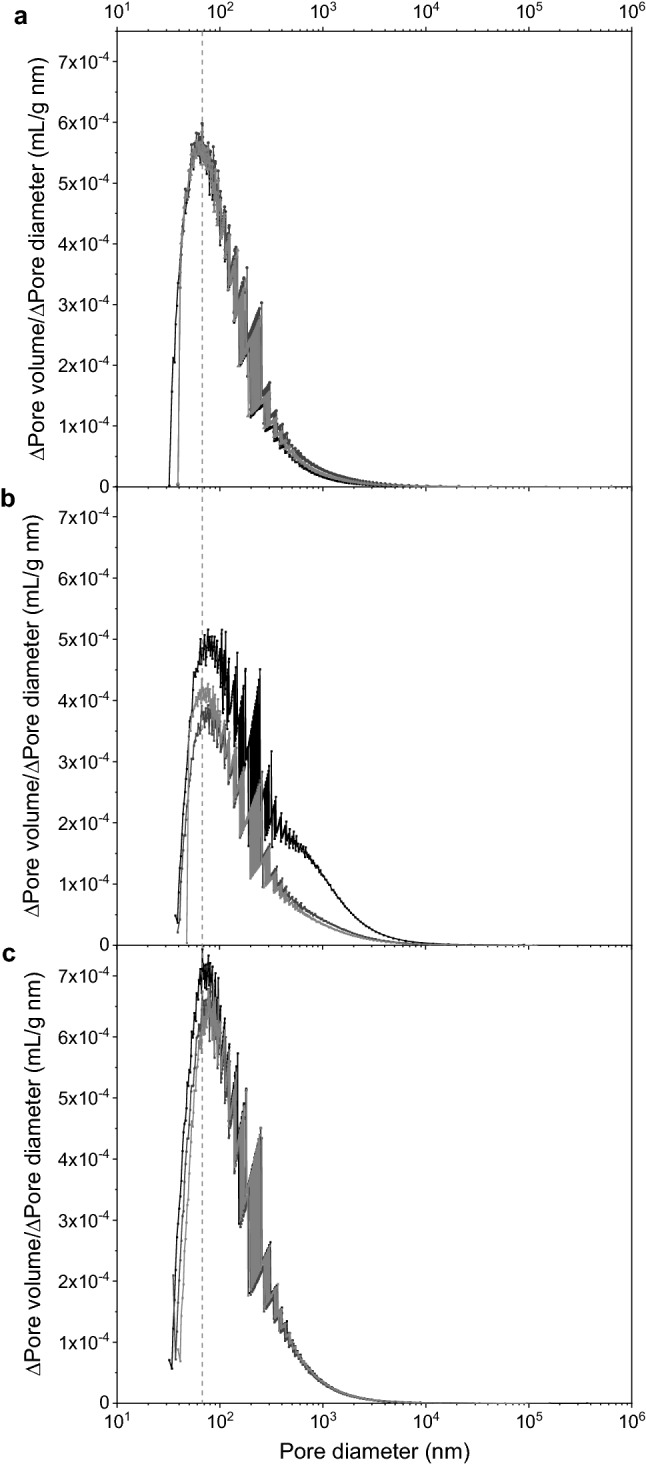
The pore volume distributions of rehydrated alginate/starch beads (a) are shifted when either formalin-casein (b) or casein (c) is added, and are broadened in case of formalin-casein. Distributions were determined by differential scanning calorimetry. Replicates (n = 3) are shown in different grey values. The dashed line marks the peak of alginate/starch beads

### Statistical analysis

Statistical analyses were conducted with SPSS Statistics v. 27 (SPSS, Chicago, USA) with a significance level of p < 0.05, except for spore density analysis of sealed and vented cultures, which were conducted with a significance level of p < 0.01. All data are presented as mean values ± standard deviations (SD). Percentage data on the water activity and the rehydration factor were arcsine transformed prior to analysis of normality and homogeneity of variance with Shapiro–Wilk's and Levene's test, respectively. To analyze the influence of the treatments or the influence of treatment and time, one-way or repeated measures (RM) analysis of variance (ANOVA) with Bonferroni post hoc tests were conducted for normally distributed and homogeneous data. Welch's correction was applied for nonhomogeneous variances. The sphericity of the matrix assumption was assessed with the Mauchley sphericity test and F values were corrected accordingly using the Greenhouse–Geisser approach*.* For non-normally distributed data sets, Kruskal–Wallis tests with multiple comparisons between groups were calculated.

## Results

### Analysis of calcium alginate/starch beads

First, we investigated plain calcium alginate/starch beads without any fungal biomass in terms of their nutrient content, water absorbency and pore structure.

Beads of different compositions appeared relatively homogeneous, rigid and did not disintegrate. The sphericity factor of dried and rehydrated beads were as follows, 0.076 ± 0.010 and 0.082 ± 0.003 for alginate/starch beads, respectively, 0.066 ± 0.003 and 0.071 ± 0.008 with FC, respectively, and 0.072 ± 0.010 and 0.065 ± 0.001 with casein, respectively (three replicates with 89–112 beads per replicate). The circularity of beads of different composition, either dried or rehydrated, was consistently between 0.88–0.89.

#### Increased C/N ratio by formalin-casein

We analyzed the formulation matrices with a C/N elemental analyzer to assess the nutritional influence of casein and FC. As expected, alginate/starch beads contained almost no nitrogen. Despite equal proportions of casein and FC in the mixture, incorporating FC, however, doubled the nitrogen content compared to casein, resulting in higher C/N ratios of 17.33 compared to 9.72, respectively (Table 1). Measurements were repeated once (Online Resource 1, Table A1).

**Table 1:** Elemental analysis of alginate/starch beads and alginate/starch beads with either casein or formalin-casein (FC) by means of an elemental analyzer

Formulation	Carbon (%)	Nitrogen (%)	C/N ratio (–)
Alginate/starch	37.05	0.03	1402.25
Alginate/starch with casein	37.93	2.19	17.33
Alginate/starch with FC	40.65	4.18	9.72

In a simple cultivation test on agar with starch and either casein or FC, we observed slightly enhanced mycelial growth for FC (Online Resource 1, Fig. A3).

**Fig. 3:**
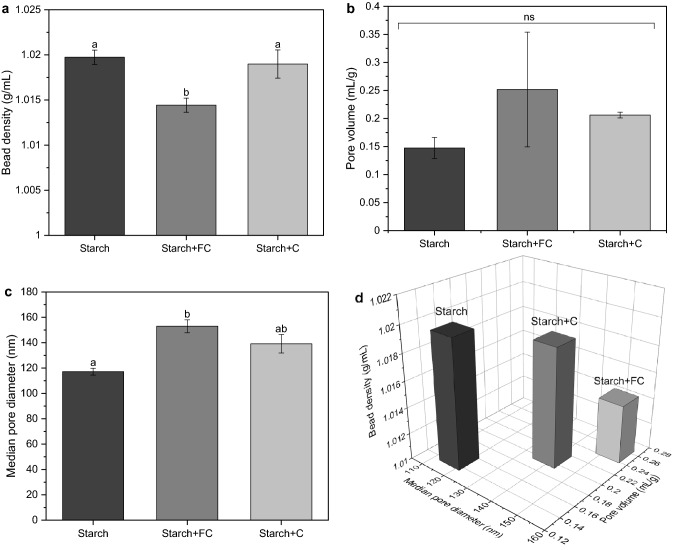
Incorporating formalin-casein (FC) into alginate/starch beads reduced the bead density (**a**) and increased the total pore volume (**b**) as well as the median pore diameter (**c**). The relationship of these three parameters becomes clear in **d**. Alginate/starch beads and alginate/starch beads with either FC or casein were rehydrated prior to analysis. Different letters above bars indicate significant differences according to ANOVA with Bonferroni post-hoc test at P < 0.05 for bead density (n = 20) and Kruskal–Wallis test at P < 0.05 for pore volume and median pore diameter (n = 3)

#### Enhanced rehydration by formalin-casein

In order to study whether casein or FC influence the water absorption and water activity of alginate/starch beads, dried beads were rehydrated on water agar over a period of 48 h.

Alginate/starch beads without or with either casein or FC were dried to a_w_ 0.174–0.175, 0.239–0.256 and 0.165–0.178, respectively. The water absorption of beads with either casein or FC differed significantly, also across time (F_2,6_ = 7.3, *P* = 0.03, F_3.2,9.8_ = 5.3, *P* = 0.02, respectively). Interestingly, casein initially decelerated the water absorption compared to alginate/starch beads with or without FC, finally reaching a rehydration factor of alginate/starch beads only (Fig. 1). In contrast, FC accelerated the water absorption after 15 min compared to alginate/starch, thus, resulting in a higher maximum rehydration factor of 0.37 ± 0.01 compared to 0.27 ± 0.00, respectively.

The bead composition and the interaction of bead composition and time also affected the water activity significantly (F_2,6_ = 256.3,*P* < 0.001, F_5.4, 16.4_ = 59.9,*P* < 0.001, respectively). Casein initially decreased water activity of beads, reaching only 0.848 ± 0.019 after 15 min, compared to 0.953 ± 0.007 for FC and 0.954 ± 0.003 for alginate/starch. Regarding rehydration factor or water activity, all formulations reached equilibrium after 2 h.

Overall, casein, initially decelerated the water absorption, and thus reached the equilibrium of starch only. FC, however, partly accelerated and finally enhanced the water absorption of alginate/starch beads.

#### Altered bead porosity and bead density

Next we investigated how casein and FC affect the internal, porous structure of the alginate/starch beads, more specifically, bead density, pore volume and pore size. The bead density of rehydrated beads was determined via settling velocity in water, and the pore volume as well as the pore size were determined by thermoporometry using differential scanning calorimetry.

The formulation composition significantly affected the bead density (F_2,59_ = 127.7, *P* < 0.001) and the median pore size (F_2,9_ = 7.2, *P* = 0.03), but not the total pore volume (F_2,9_ = 4.62, *P* = 0.10).

The pore volume distributions revealed a broader spectrum of pores for FC with more pronounced shoulders and peaks were visibly shifted to larger pore sizes for both casein and FC (Fig. 2). Consequently, both casein and FC increased the median pore diameter by 19 and 31%, respectively (Fig. 3). The results on pore volume, determined by thermoporometry, were not significant, but there was a slight trend towards larger pore volume with FC, which is supported by the findings on bead density. In contrast to casein, FC lowered the bead density of alginate/starch beads significantly from 1.02 ± 3.1 10^–7^ g/mL to 1.014 ± 2,8 10^–7^ g/mL (F_2,57_ = 127.753, *P* < 0.001). Additional data are given in Online Resource 1 Table A2.

In sum, FC had a greater impact on porosity and density than casein, resulting in less dense beads with larger pores.

### Assessment of the fungal activity

Next, we assessed the activity of either *M. brunneum,* or both *S. cerevisiae* and *M. brunneum*, in Kill beads or A&K beads, respectively, by determining the spore formation and CO_2_ release.

#### Enhanced sporulation of *Metarhizium brunneum*

**Fig. 4:**
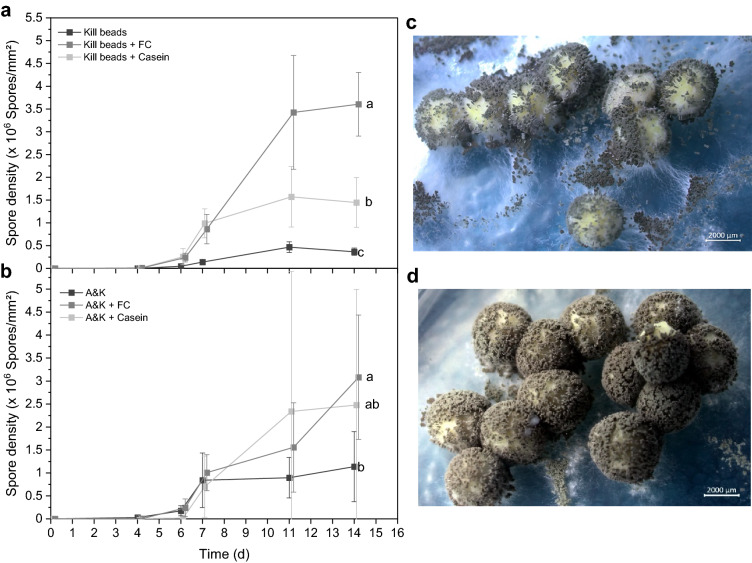
Formalin-casein (FC) significantly enhanced the spore formation on both Kill beads (**a**) and A&K beads (**b**) compared to beads containing only starch and even compared to Kill beads containing casein. Beads were rehydrated and cultivated on 1.5% water agar at 25 °C. Different letters indicate significant differences according to RM-ANOVA with Bonferroni Post hoc test (n = 10). Spore density on A&K beads (**c**) was visibly enhanced by formalin-casein (**d**) after 17 d

We asked, whether casein or FC impact the spore formation of *M. brunneum* on beads. Moreover, it should be investigated to what extent a possible effect can be observed on beads containing additionally encapsulated *S. cerevisiae*. Therefore *M. brunneum* was individually encapsulated (Kill beads) or co-encapsulated with *S. cerevisiae* (A&K beads) in alginate/starch beads with either casein or FC. Beads were dried to a_w_ 0.29–0.46. Both casein and FC increased spore density on Kill and A&K beads, but only FC led to a significant difference on both (F_2,27_ = 4.0,*P* = 0.03; F_2,27_ = 116.4, *P* < 0.001) and also across time (F_2,27_ = 4.3,*P* = 0.02; F_3.6,48.3_ = 28.7,*P* < 0.001) (Fig. 4). Accordingly, maximum spore density on Kill and A&K beads was enhanced by FC by the factor 7 and 3, respectively. Casein, however, showed strong differences between replicas. On the one hand, strong mycelial growth and sporulation was observed on the beads, but on the other hand, there were beads with almost no mycelial growth or even excessive yeast growth. After 7 days, the spore forming rate of *M. brunneum* was particularly enhanced by FC, irrespective of whether *S. cerevisiae* was co-encapsulated. Incorporating FC did not accelerate the onset of spore formation but increased the spore density on both Kill-beads and A&K beads. Nevertheless, the impact was more pronounced when *M. brunneum* was encapsulated individually. Overall, FC had a higher impact than casein, since incorporating casein showed inconsistent results.

Increasing the FC content and omitting starch concurrently, did not increase or fasten the spore formation, in contrast, it significantly reduced the spore density over time, presumably due to the lack of carbon (Online Resource 1, Fig. A4).

**Fig. 5:**
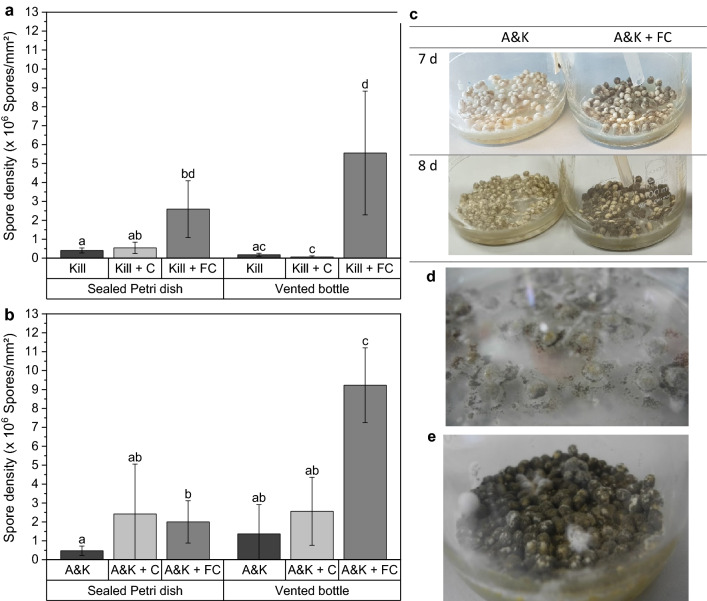
Aeration significantly increased spore density exclusively when formalin-casein (FC) is incorporated in Kill beads (**a**) or A&K beads (**b**). Beads were rehydrated and cultivated on 1.5% water agar either in sealed Petri dishes at 25 °C, or in vented glass bottles at room temperature. Different letters above bars indicate significant differences according to Kruskal–Wallis-test with p < 0.01 (n = 10). Spore formation was visibly accelerated on A&K beads by FC when rehydrated and cultivated in vented glass bottles, as can be seen after 7 and 8 days (**c**). Compared to the cultivation in sealed Petri dishes (**d**), aeration visibly enhanced spore yield of A&K beads containing FC (**e**)

For studies on CO_2_ release (see ‘CO_2_ release from beads’) the different Kill and A&K formulations from the same batch previously described, were aerated during cultivation. The sporulation differed quite obviously in the course of the experiment and it became apparent that to some extent spores were formed earlier (Fig. 5). Therefore, the question arose, whether there is an interaction between aeration and the formulation additives. Thus, spore densities of beads cultured in sealed Petri dishes or in vented glass bottles were determined after 21 days.

By aeration, the spore density was increased on both Kill beads and A&K beads but only when FC was incorporated (Fig. 5). As a result, the spore density on A&K beads with FC was significantly increased more than fourfold from (2.0 ± 1.1)·10^6^ spores/mm^2^ to (9.2 ± 2.0)·10^6^ spores/mm^2^ (H_5_ = 51.509, *P* < 0.001).

Taken together, incorporating FC and subsequently venting the cultures increased the spore density even by the factor 18. Surprisingly, aeration had a negative impact on spore formation on Kill beads when casein was added (from 5.4 ± 3.0 10^5^ to 0.5 ± 0.5 10^5^ spores/mm^2^). However, this was not seen for A&K beads, confirming the inconsistency of results mentioned previously. Overall, enhanced mycelial growth could be observed to some extent, but a positive and significant change in sporulation due to aeration was observed exclusively for FC. These results were in line with the drying kinetics of alginate/starch beads, which showed faster drying when FC was incorporated (Online Resource 1, Fig. A5).

#### Enhanced CO_2_ release from beads

To evaluate how casein and FC influence the respiratory activity of *M. brunneum* and *S. cerevisiae* and hence the CO_2_ release from beads, two different bead formulations namely Kill beads containing only *M. brunneum* and A&K beads containing both fungi, *M. brunneum* and *S. cerevisiae,* were examined.

**Fig. 6:**
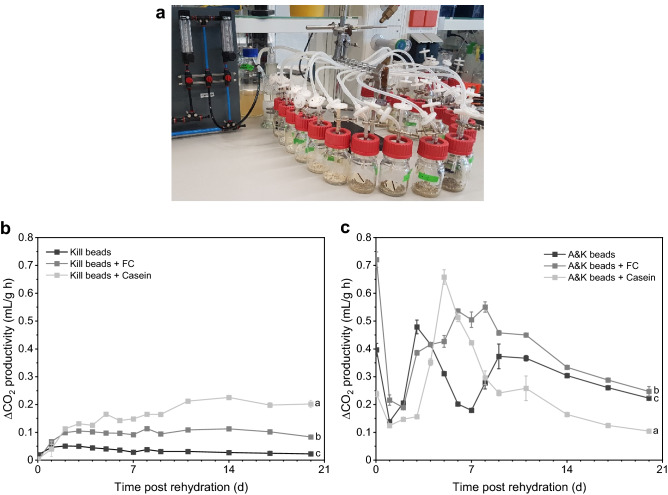
Experimental set-up (**a**) for CO_2_ release measurements. Beads were rehydrated and cultivated on 1.5% water agar at room temperature in vented glass bottles at 15 L/h (one volume exchange per 10 min) using a variable area flowmeter and a compressed air distributor. Formalin-casein (FC) significantly increased the CO_2_ productivity of *M. brunneum* and *S. cerevisiae* in Kill beads (**b**) or in A&K beads (**c**) over time compared to beads without or with casein. Different letters indicate significant differences according to RM-ANOVA with Bonferroni post hoc test at P < 0.05 (n = 3)

The bead composition significantly influenced the CO_2_ productivity of both Kill and A&K beads (F_2,6_ = 1708.9, *P* < 0.001 and F_2,6_ = 271.7, *P* < 0.001, respectively, Fig. 6). Accordingly, the Kill beads CO_2_ production increased twofold within the first 2 days with either casein or FC compared to plain Kill beads. Over the course of 5 days a greater influence by casein on the Kill beads was revealed, resulting in a doubled and even tenfold CO_2_ productivity compared to Kill beads with or without FC after 10 days. The total amount of CO_2_ produced by *M. brunneum* was five-folded from 16 g/L to 81 g/L by casein and three-folded to 47 g/L by FC.

Plain A&K beads reached their maximum productivity fastest, after 3 days, but collapsed also, recovering after 4 days. The CO_2_ productivity was slightly delayed when casein was incorporated into A&K beads, but peaked at 0.65 mL/g h after 5 days. Nevertheless, CO_2_ production also collapsed subsequently. In contrast, FC led to consistent production of CO_2_ across time, mitigating the negative effect, observed for plain A&K beads. Therefore, A&K beads with FC provided a higher CO_2_ release over a longer period of time. Moreover, FC enhanced the total CO_2_ produced by *M. brunneum* and *S. cerevisiae* in 20 days by 29% (from 142 g/L to 183 g/L), whereas casein even decreased it by 19% (from 142 g/L to 115 g/L). Thus, A&K beads containing casein produced only 63% of the CO_2_ that A&K beads containing FC released.

The replica confirmed these surprising results and FC increased the total CO_2_ yield even by 34% from 92 mL/g to 123 g/L (Online Resource 1, Fig. A6). FC increased the CO_2_ productivity of *M. brunneum* and probably *S. cerevisiae* over a period of 20 days.

Even though incorporating FC did not reach the maximum CO_2_ productivity, it led to a more continuous production and hence to an increased total CO_2_ release.

We investigated whether the fluctuations in CO_2_ productivity of plain A&K beads were somehow caused by fluctuating water content or water activity. Beads were placed on semi selective water agar in Petri dishes and measured daily for 8 days. However, water absorption remained unchanged after 24 h for all formulations as they reached maximal water activity possible, which equaled that of moist beads (Online Resource 1, Fig. A7).

## Discussion

In this study, we found that FC influences the porous properties of alginate/starch beads, thus enhancing their water absorbency. Moreover, FC increases the fungal activity of encapsulated *M. brunneum* and *S. cerevisiae*, leading to enhanced spore formation and CO_2_ productivity.

### C/N ratio

To assess the nutritional influence of FC, non-crosslinked casein was used as reference. Since both casein formulations differed only slightly in their carbon content, but noticeably in their nitrogen content, different impacts on sporulation must be taken into account. It is well known that type and concentration of carbon and nitrogen sources as well as the C/N ratio impact growth and sporulation kinetics of fungi (Ajdari et al. 2011). In a study of Li and Holdom (1995), soluble starch supported growth and sporulation of *Metarhizium anisopliae (M. anisopliae)*, and according to Braga et al. (1999) casein even reduced the lag phase and also increased the growth rate of *M. anisopliae* (Braga et al. 1999; Li and Holdom 1995). In the present study, incorporating casein led to a higher C/N ratio compared to FC. *M. anisopliae* favored higher C/N ratios of 160, when sucrose as carbon and yeast extract as nitrogen source were used (Gao and Liu 2010), assuming that casein should offer more favorable nutritional conditions than FC.

### Rehydration vs. bead density, pore volume distribution and median pore size

Rehydration kinetics were significantly influenced by casein and FC. The water absorbency was increased only by FC, reaching higher rehydration factors. However, the water activity was not enhanced and was even decreased by casein within the first hours. As the water activity reached its maximum, the water absorption still increased, thus, the porous structure of rehydrated beads was examined. Rehydrated beads with higher pore sizes should have shown a higher rehydration rate, which applies for FC but not for casein. The decreasing bead densities as well as the increasing median pore sizes and the tendency towards larger pore volumes are mutually supportive and strong indicators for the involvement of casein in the increased water absorption, although results were not as pronounced when casein was added. Rehydration involves different physical mechanisms such as capillary flow, convection and diffusion (Vreeker et al. 2008). According to Caccavo et al. (2018), the water transport in gel phases is complex and mainly driven by diffusion processes which however do not follow a pure Fickian behavior most of the time (Caccavo et al. 2018). FC, which is primarily investigated as tablet disintegrant, hardly swells during tablet disintegration. Non swelling agents are believed to impart their disintegration action through increased porosity and capillary action (Mohanachandran et al. 2011; Ponchel and Duchene 1990). Larger pore diameters result in faster capillary action with decreasing speed over time (Andersson et al. 2017). In this study, the latter is reflected by the rehydration factor which was increased by FC and decelerated over time. The mechanisms of capillary action in hydrogels are mainly based on water-water interaction. The reason why capillary flow even occurs is that water tends to lower its surface energy, by filling the pores with water so that the surface area exposed to air is reduced (Andersson et al. 2017). In contrast to FC, the water absorption of beads containing casein was decelerated compared to beads containing only starch, although the median pore size and bead density did not change. There is no explanation for this so far.

Overall, the pore size may be underestimated due to a considerable fraction of non-freezable water interacting with alginate/starch/casein, which is difficult to determine for hydrogels because the non-freezable water could exist not only on the pore surface but also in the matrix (Kazuhiko Ishikiriyama et al. 1995a, b). We assumed that this fraction is similar for each formulation, and may be assumed to equal 1.0 nm (Ishikiriyama et al. 1995a, b), and thus, determined pore sizes should be comparable. Moreover, the beads needed to be cut to fit into the aluminum pans, and to not exceed maximal weight. The smaller the sample size, and the bigger the contact area with the pan, the better the heat flux and the more accurate the measurement. Cutting surely disorganizes the porous structure, at least in the cutting zone, but we assumed that the potential error is smaller compared to that caused by a modified production process making microbeads. Results obtained from thermoporometry by DSC especially for polymers must be considered carefully and viewed with caution, because they can be affected by measurement conditions, which can cause e.g. water migration. Consequently, measurements made on heating can underestimate pore sizes whereas measurements made on cooling can overestimate them (Hay and Laity 2000; Iza et al. 2000). However, pore size distributions of polymer membranes determined by thermoporometry were concluded to be valid and it was made clear that thermoporometry is a unique and the most reliable method to characterize mesoporous structures of hydrogels and pore sizes of polymer membranes, especially since it provides several advantages where classic methods such as mercury porometry fail or are limited to dry samples (Hay and Laity 2000; K. Ishikiriyama et al. 1995a, b; Iza et al. 2000; Riikonen et al. 2011). In the present study, the observation that results of replicates were quite consistent, strengthen the methods validity. The influence of the initial porosity of the dried system is of great interest, which however warrants a different methodology and may be subject of further investigations.

Rehydration experiments in this study were conducted under optimal conditions with maximum water availability, which does not reflect field conditions. Further studies under suboptimal conditions could reveal a stronger rehydration effect of FC. Moreover, rehydration depends on the ionic strength of the rehydration medium (Fang et al. 2011), thus, alginate barely swells in pure water. Its rehydration depends on the electrolyte solutions and increases with increasing salt concentrations, until disintegration occurs (Vreeker et al. 2008). Adding salts also influences the water binding by proteins because of their implications on electrostatic interactions (Zayas 1997). In this study, beads were rehydrated in ultra-pure water, so it can be assumed that water absorption is further favored in other media or by soil water during field application.

### Activity of *Metarhizium brunneum* and *Saccharomyces cerevisiae*

FC enhanced the activity of *M. brunneum* more than casein, as revealed by increased sporulation. In view of our observation on pore structure and water absorption, we attribute the increased spore density to the altered network structure caused by FC. The effect was even more pronounced when the system was aerated, although beads were cultivated at a lower temperature (23 °C instead of 25 °C) due to logistic reasons, and *M. brunneum* growth is known to be temperature dependent. This indicates that gas exchange like oxygen supply plays an important role which might be favored in the more porous structure conveyed by FC.

Interestingly, FC’s effect on spore yield decreased once *S. cerevisiae* was co-encapsulated, whereas casein’s impact was slightly improved on average, though spore yields were inconsistent. It is conceivable that *S. cerevisiae* compensated for the influence of FC on the porous structure due to its relatively high proportion in the matrix. We observed, that *S. cerevisiae* proliferation was favored on/in beads containing casein. However, stronger proliferation of *S. cerevisiae* coincided with poor sporulation of *M. brunneum*. There might have been an interaction between casein and *S. cerevisiae*, which led to inhibition of spore formation. It has already been reported that yeast, among them *S. cerevisiae*, are capable of decreasing the growth of filamentous spoilage fungi (Armando et al. 2012; Petersson and Schnürer 1995) and that actively growing yeast may affect the development of other microorganisms by acidifying the environment (Walker 1998), or by competing for space and nutrients (Armando et al. 2012). Moreover, active *M. brunneum* and *S. cerevisiae* may compete for oxygen as oxygen consumption rises. *M. brunneum* conidia production was increased by aeration, but only when FC was incorporated, which suggests that FC has a major influence on the alginate network and thus on oxygen diffusion, which impacts the proliferation of both fungi (Braga et al. 1999; Gosmann and Rehm 1986). Garcia-Oritz et al. (2015) found that the conidial production of *M anisopliae* is enhanced and accelerated by oxygen-enriched pulses without impairing conidia quality (Garcia-Ortiz et al. 2015).

Moreover, it must be noted that the nutritional values of alginate/starch beads were measured only. In A&K beads the impact of (dead) *S. cerevisiae* as nutrient supply must be considered, since yeast cells are rich nutrient sources, comprising 40–60% of their biomass protein (Walker 1998). It is worth mentioning that nutritional conditions can alter the germination speed, adhesion and pathogen virulence of aerial conidia (Rangel et al. 2008), which should be considered in virulence tests and field trials.

Overall, we have not yet resolved, how the fungus develops inside the beads and how growth is influenced, since we investigated sporulation only.

It is known that co-encapsulating *S. cerevisiae* and the entomopathogenic fungus *Beauveria bassiana* enhances the CO_2_ release of moist alginate/starch beads compared to the respective single encapsulation (Vemmer et al. 2016), which is in line with our findings and emphasizes the importance of co-encapsulating *S. cerevisiae.* By adding FC, CO_2_ was produced more constantly compared to regular A&K beads, containing only starch. Why the CO_2_ productivity of these beads collapsed between 4 and 7 days remains unclear, but both caseins seemed to mitigate the negative effect. In the study of Vemmer et al. (2016) no decrease in CO_2_ productivity has been observed when *B. bassiana* was encapsulated, indicating a possible impact of *M. brunneum*. However, it must be noted that moist beads were investigated in their study. Despite, in our study, maximum CO_2_ was produced faster, which is in line with Humbert et al. (2017) who also studied, the CO_2_ release of dried alginate/starch beads, but depending on amyloglucosidase content. There, maximum CO_2_ concentration was reached after 7 days. This is in line with our results obtained from A&K beads containing casein or FC. However, fluctuations in CO_2_ concentrations were not observed, even in the absence of amyloglucosidase (Humbert et al. 2017b).

Taking these studies together, there is nothing to indicate that drying and rehydration had any negative effect or, that amylase was lacking, when *M. brunneum* was encapsulated. It rather suggests that there is complex interaction between the amylase producing *M. brunneum* and the CO_2_ producing *S. cerevisiae*, which might also be nutrient mediated by casein. Furthermore, visible spore formation coincided with highest CO_2_ productivity of A&K beads containing FC. The increased maximal CO_2_ release by FC coincided with enhanced conidia production, thus, better attraction of wireworms within the first week and enhanced probability of killing may possibly be achieved.

There are two major limitations of this study. Although non-crosslinked casein was used as reference, we cannot rule out an additional nutritive impact of FC, since casein and FC slightly differed in their nutritional content. However, adjusting the FC content to equal the nutritional content of casein, might also change the bead structure, thus, one cannot be considered separately from the other.

Secondly, we could not determine the porous properties of Kill beads or A&K beads and thus, we can only assume, that the impact of FC on the bead structure is similar. Determining the pore volume distribution and pore sizes by thermoporometry requires salt-free samples, since the measuring principle is based on the melting point depression of pore water and can thus interfere with the measurement. More work has to be done to implement a valid method for alginate beads containing living cells. Nevertheless, the measurements regarding pore size and the impact of aeration on fungal activity are strong indicators for an altered bead structure that should be confirmed by other methods such as SEM.

In conclusion, FC influenced the porous properties of alginate/starch beads, possibly by implementing an altered network structure, thus enhancing their water absorbency. Moreover, FC increased the fungal activities of encapsulated *M. brunneum* and *S. cerevisiae*, resulting in enhanced CO_2_ productivity and increased spore yields.

Our data suggest that FC enhances the oxygen diffusion through modified pore structure, since aeration enhanced the fungal development. It is recommended to verify the altered oxygen diffusion using e.g. oxygen microelectrodes. Moreover, enlightening the complex symbiotic interaction of *M. brunneum* and *S. cerevisiae* is of great interest to further adjust the bead formulation. The obtained findings can be applied to other biological crop protection products, especially other microbial pest control agents.

## Supplementary Information

Below is the link to the electronic supplementary material.

Supplementary file1 (PDF 267 kb)
