# Supplementary material for: Formalin-casein enhances water absorbency of calcium alginate beads and activity of encapsulated Metarhizium brunneum and Saccharomyces cerevisiae
Source: World J Microbiol Biotechnol. 2021 Aug 18;37(9):156. doi: 10.1007/s11274-021-03121-3 (PMC8373754; doi:10.1007/s11274-021-03121-3)
Supplement: Supplementary file 1 — Supplementary file1 (PDF 267 kb) [file 11274_2021_3121_MOESM1_ESM.pdf]

World Journal of Microbiology and Biotechnology

Formalin-casein enhances water absorbency of calcium alginate beads and activity of encapsulated *Metarhizium brunneum* and *Saccharomyces cerevisiae*

Katharina M. Hermann[1,2], Alexander Grünberger[2], Anant V. Patel[1,*]

[1] Faculty of Engineering and Mathematics, Fermentation and Formulation of Biologicals and Chemicals, Bielefeld University of Applied Sciences, Bielefeld, Germany

[2] Faculty of Technology, Multiscale Bioengineering, Bielefeld University, Bielefeld, Germany

[*]Corresponding author:

Anant V. Patel

Bielefeld University of Applied Sciences, Faculty of Engineering and Mathematics, Fermentation and Formulation of Biologicals and Chemicals, Interaktion 1, 33619 Bielefeld

Phone number: +49 521 106 7318

Email: anant.patel@fh-bielefeld.de

Supplementary information

```
2 input = getDirectory("Choose a Directory");
3
4 output = input+"output"+File.separator;
5 File.makeDirectory(output);
6  if (!File.exists(output))
7   exit("Unable to create directory");
8
9 list = getFileList(input);
10 count = newArray;
11 filenames = newArray;
12
13 for (p = 0; p<list.length; p++) {
14 if (endsWith(list[p], ".JPG") || endsWith(list[p], ".jpeg") || endsWith(list[p], ".jpg") || endsWith(list[p], ".TIFF")) {
15 file=list[p];
16 open(input+File.separator+file);
17
18 makeLine(928, 1844, 1230, 1817);
19 run("Set Scale...", "distance=303.2344 known=10 pixel=1 unit=mm global");
20
21 run("Gaussian Blur...", "sigma=3");
22 run("8-bit");
23
24 //run("Threshold...");
25 //setThreshold(172, 255);
26 setOption("BlackBackground", false);
27 run("Convert to Mask");
28 run("Watershed");
29 //setTool("oval");
30 makeOval(464, 32, 2657, 2732);
31 run("Analyze Particles...", "size=2-50 circularity=0.75-1.00 show=Overlay display exclude clear add");
32 run("Read and Write Excel");
33 saveAs("JPG",output+File.separator+file+"_HM.tif");
34
35 run("Close All");
36 run("Clear Results");
37 }}
```

Fig. A1 Exemplary macro used for counting beads and measuring the bead size batchwise using Fiji (ImageJ, U. S. National Institutes of Health, Bethesda, USA). Images from one folder are analyzed, and masked images are saved in a new folder and the results are exported to an excel spreadsheet. Following parameters need to be customized for each batch. In lines 18 and 19 the global scale is set which refers to the scale shown in the image. In line 25 the threshold is set which depends on the exposure during shooting. In line 30 only the section the size of the petri dish in which beads are placed is considered for measurements. In line 31 the specific and estimated particle parameters need to be adjusted.

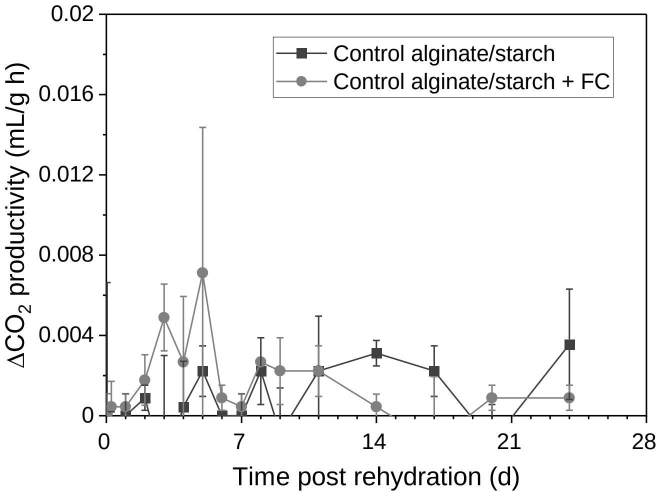

Fig. A2 The CO_2 productivity of control alginate/starch beads, without (squares) or with formalin-casein (FC, circles) both without biomass, was negligibly low (n=3)

Table A1 Elemental analysis of alginate/starch beads and of alginate/starch beads with either casein or formalin-casein by means of an elemental analyzer. Each formulation was measured twice

No.	Formulation	Carbon (%)	Nitrogen (%)	C/N ratio (-)	Sulphur (%)	Hydrogen (%)
1	Alginate/starch	37.05	0.03	1402.25	0.053	6.123
2	Alginate/starch	37.10	0.03	1381.83	0.046	6.162
1	Alginate/starch with casein	37.93	2.19	17.33	0.260	6.113
2	Alginate/starch with casein	38.09	2.17	17.52	0.248	6.164
1	Alginate/starch with FC	40.65	4.18	9.72	0.366	6.315
2	Alginate/starch with FC	40.76	4.22	9.67	0.374	6.321

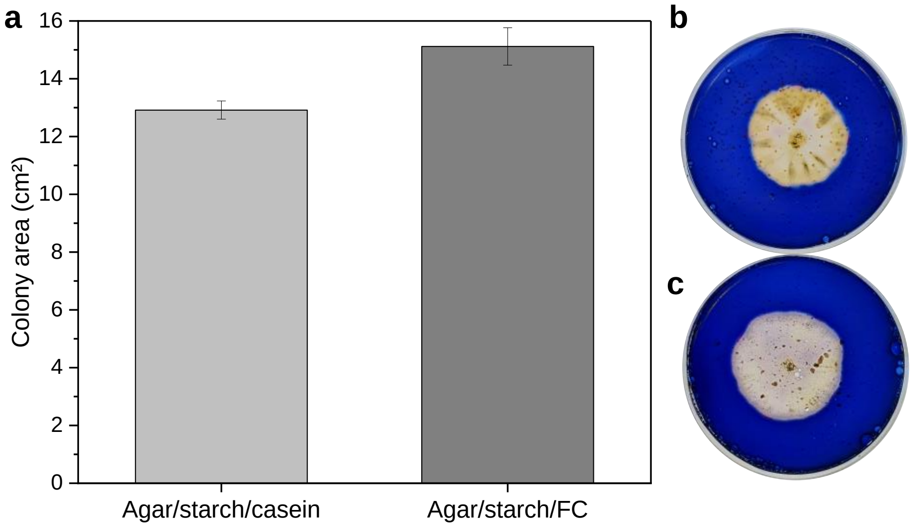

Fig. A3 Formalin-casein (FC) slightly increased *M. brunneum* colony size after 14 days (a). Filtered and washed mycelium from liquid culture was placed on agar plates (15 g/L agar, 10 g/L soluble starch, 5 g/L formalin-casein or casein) and was cultivated at 23 °C in the dark. After 14 d, plates were flood with Lugol's iodine solution to assess amylase activity. No clear lysis zones were detected around the colonies, neither for casein (b) nor for FC (c), but the colony aeras were determined by image analysis using the open source image processing program Fiji (n=4–5)

Table A2 Bead density, median pore diameter and total pore volume of alginate/starch beads containing FC or casein

Formulation	Bead density (g/mL)		Median pore diameter (nm)		Total pore volume (mL/g)	
	MV*	SD	MV*	SD	MV*	SD
Alginate/starch	1.0197 a	0.0008	117.1 a	2.7	0.147 a	0.019
Alginate/starch with FC	1.0144 b	0.0008	153.0 b	5.1	0.252 a	0.102
Alginate/starch with Casein	1.0189 a	0.0016	139.1 ab	7.3	0.206 a	0.005

* Different letters behind values indicate significant differences according to Kruskal-Wallis test at $P<0.01$ (n=3)

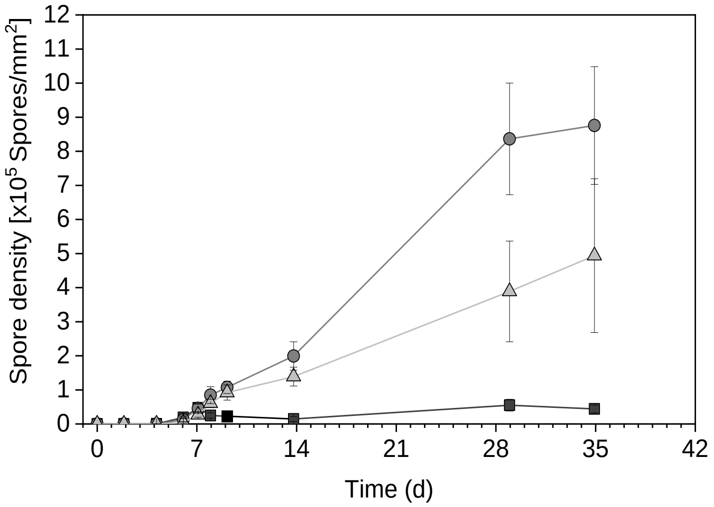

Fig. A5 Incorporating formalin-casein in A&K beads and omitting starch (squares) decreased the spore density, whereas the combination of starch and formalin-casein (circles) enhanced it compared to regular A&K beads (triangles). Beads were rehydrated and cultivated on 1.5 % water agar in Petri dishes at 25 °C in the dark

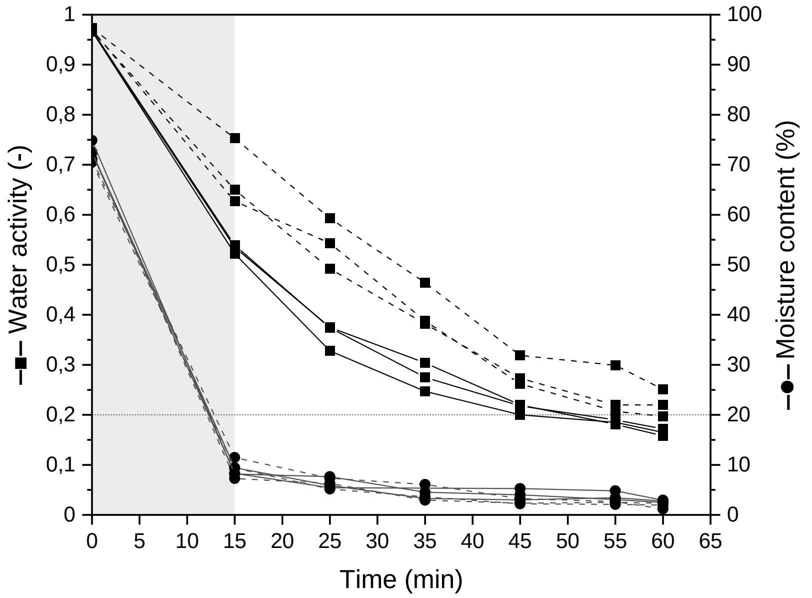

Fig. A2 Formalin-casein accelerated the drying process of alginate/starch beads. For each run, an amount of 170 g moist alginate/starch beads (dashed lines) with formalin-casein (solid lines) were dried at 60 °C for 15 min (indicated by grey background) and at 40 °C for 45 min in a self-constructed fluidized bed dryer. The water activity (squares) was determined by means of a water activity meter and the moisture content (circles) was measured with a moisture analyzer

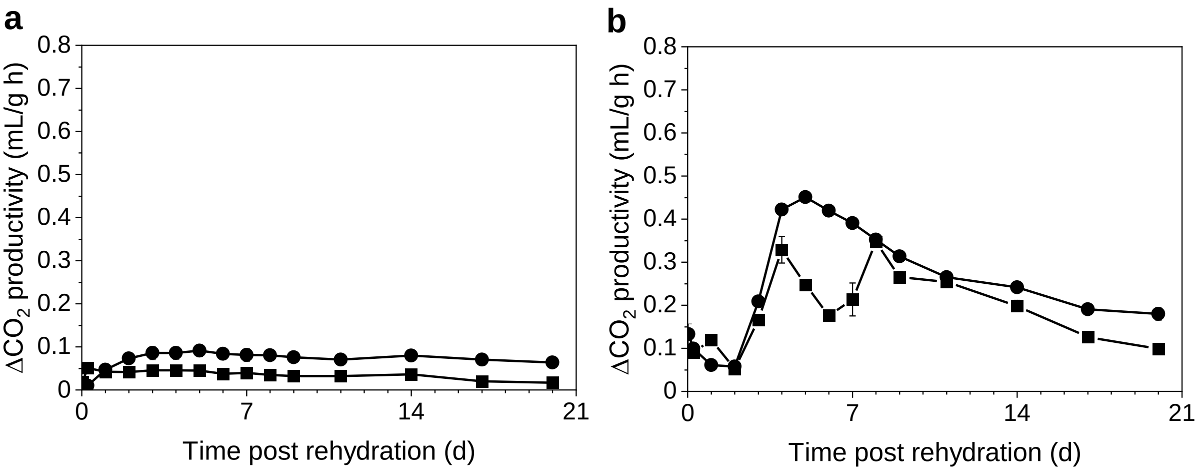

Fig. A6 Verification of CO_2 productivity of Kill beads (a) and A&K beads (b) each without (squares) and with formalin-casein (circles). Beads were rehydrated and cultivated on 1.5 % water agar in vented glass bottles at room temperature (n=3)

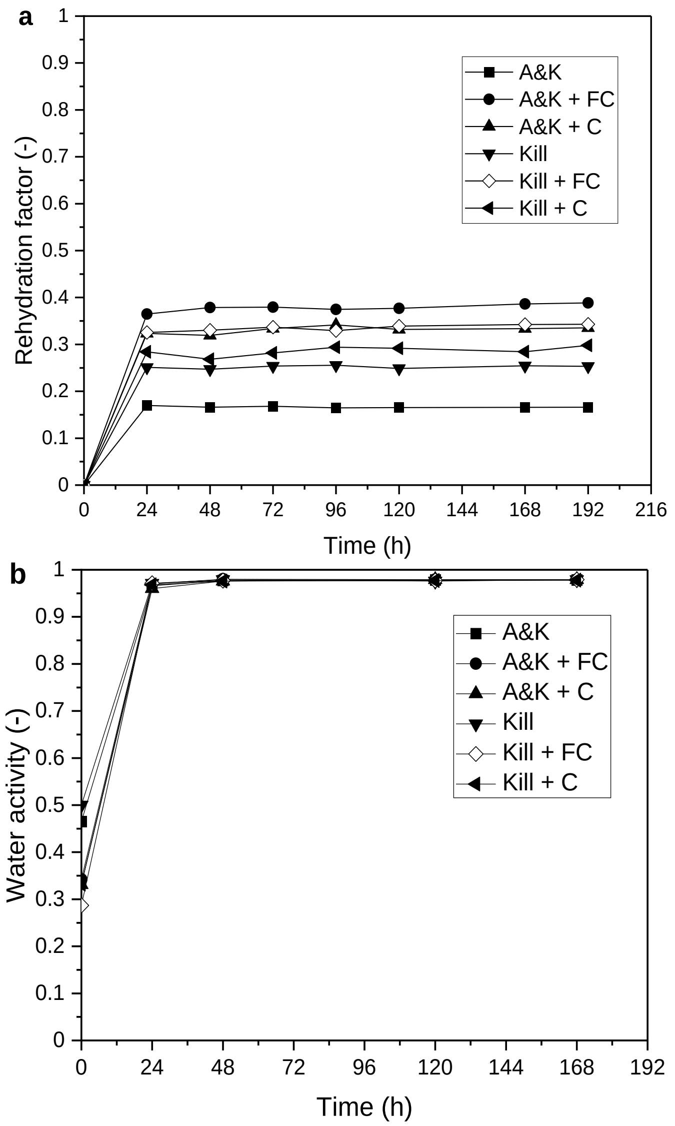

Fig. A3 The rehydration factors (a) and the water activities (b) of different formulations reached maximum after 24 h and did not fluctuate over the course of 8 days and 7 d, respectively. Beads were rehydrated on 1.5 % water agar containing antibiotics and fungicides to suppress the growth of *M. brunneum* and *S. cerevisiae* and to avoid contaminations
